# Empirical treatment for TB in HIV: lessons from a cohort study of people living with HIV treated in Recife, Brazil

**DOI:** 10.1186/1471-2458-14-289

**Published:** 2014-03-29

**Authors:** Maria de Fátima Pessoa Militão Albuquerque, Isabella Coimbra, Joanna d’Arc Batista, Magda Maruza, Ricardo A A Ximenes, Heloísa R Lacerda, Demócrito B Miranda-Filho, Marcela L Santos, Laura C Rodrigues

**Affiliations:** 1 NESC Department, Centro de Pesquisas Aggeu Magalhães/FIOCRUZ, Recife, Brazil; 2 Department of Medical Science, Universidade de Pernambuco, Recife, Brazil; 3 Department of Tropical Medicine, Universidade Federal de Pernambuco, Recife, Brazil; 4 Hospital Correia Picanço, Recife, Brazil; 5 London School of Hygiene and Tropical Medicine, London, UK

**Keywords:** TB/HIV, Mortality rate, Survival analysis

## Abstract

**Background:**

Tuberculosis (TB) is the leading cause of death related to HIV worldwide. This study analyzes the survival of People Living with HIV (PLHIV) reporting cough without bacteriological confirmation of TB and identify factors associated with death.

**Methods:**

Prospective cohort with a consecutive sample of PLHIV, aged ≥ 18 years. Patient inclusion criteria were complaint of current cough of any duration at the time of the first study interview or during their subsequent routine visits to health services and for whom AFB sputum smear was either negative or not performed during the whole follow-up period. Kaplan-Meier method was used to calculate the probability of survival. We estimated the Hazard Ratio (HR) in bivariate and multivariate Cox regression analyses.

**Results:**

Mortality was 4.6 per 100 py; 73% were receiving HAART at recruitment. Average time from the first recorded date of cough until empirical treatment for tuberculosis was six months. Mortality was higher when the CD4 count was low (HR = 5.3; CI 95%: 3.2-9.0; p = 0.000), in those with anemia (HR = 3.0; CI 95%: 1.6-5.6; p = 0.001) and with abnormal chest X-rays (HR = 2.4; CI 95%: 1.4-4.0; p = 0.001). Mortality was higher in those receiving empirical TB treatment (HR = 2.4; CI 95%: 1.4-4.0; p = 0.002), but only in those with normal X-rays, no history of tuberculosis and no bacteriology requests. Empirical treatment for TB was more frequent in PLHIV with low CD4 counts, anemia, history of opportunistic infections, weight loss, previous tuberculosis, negative bacteriology test (as opposed to not having a test) and abnormal chest X-ray.

**Conclusions:**

Higher mortality in PLHIV reporting a current cough without bacteriological confirmation of tuberculosis was identified for those with a CD4 cell count <200, abnormal chest X-ray, anemia and empirical treatment for tuberculosis. Mortality was not significantly higher in those empirically treated for TB, who had three characteristics suggestive of the disease (abnormal chest X-ray, history of TB treatment, AFB sputum smear or *M.tb* culture testing). Routine cohorts are not an adequate setting to evaluate the impact of empirical treatment for TB on the mortality of PLHIV.

## Background

The widespread use of HAART has caused a decline in mortality rates in PLHIV [1], although rates are still higher in developing countries than in developed countries [2]. Population surveys and autopsy studies have shown that many of these deaths are due to undiagnosed and therefore untreated tuberculosis (TB) [3-5]. TB is the leading cause of death related to HIV worldwide [3,6-9] contributing to an estimated 24% of deaths [1], and this alone may prevent meeting the WHO target of halving the 1990 levels of TB mortality [6]. Simultaneous antiretroviral and TB treatment has reduced mortality of dual TB/HIV cases in observational studies [10-12] and controlled trials [13] and is recommended by WHO [6]. However, in the severely immune-suppressed it is difficult to diagnose tuberculosis or even to establish criteria for suspecting active disease.

In developing countries, the diagnosis of pulmonary TB is heavily dependent on AFB sputum smear microscopy, which has low sensitivity among PLHIV [14], who often present extra pulmonary or sputum smear-negative pulmonary TB, resulting in late diagnosis [15] and therefore either delayed or no treatment [16]. This has led to the suggestion that empirical treatment of tuberculosis (i.e. without microbiological confirmation) may be desirable in some circumstances, given the existing limitations for the diagnosis of negative AFB sputum smear pulmonary TB in PLHIV, and the high rates of TB mortality in this population [3]. In spite of this, there has been little research into the outcomes of HIV-positive TB suspects [17]. Moreover, in the routine of health services, attending physicians may not always follow standardized protocols and it remains unclear as to how these patients are selected for empirical TB treatment.

Recife, the capital of Pernambuco, Northeast Brazil, has the 4th highest rate of TB (92/100.000 inhabitants in 2011) in the country, and high mortality rates from TB (6.1/100.00 inhabitants in 2011) [18]. In a routine care setting in Recife, we estimated the probability of survival and the mortality rate of PLHIV complaining of a cough, since this is the main symptom for suspected TB, as recommended by the Brazilian Ministry of Health) [18]. The presence of a cough of any duration is one of the four symptoms recommended for TB screening amongst PLHIV [19]. We also investigated factors associated with death, with emphasis on empirical treatment for tuberculosis.

## Methods

From an original cohort of PLHIV attending two HIV referral health centers in Recife, recruited from 2007 to 2010 and followed up to June 2011, we selected a consecutive sample of individuals who reported a cough in order to estimate the probability of survival and mortality rates. We also identified the factors associated with death with emphasis on empirical TB treatment. The patient inclusion criteria were: aged ≥ 18 years, and complaining of a current cough of any duration at the time of the first study interview, or during their subsequent routine visits to health services, and for whom AFB sputum smear examination or *Mycobacterium tuberculosis* (*M.tb*) culture tests were either negative or not performed (either because the attending physician did not request the test or because the patient did not have sputum or simply did not perform the requested tests) during the whole follow-up period.

We excluded those with positive bacteriology results (sputum smear microscopy or culture for *M.tb*) because they were confirmed TB cases. Cough was used in the study to indicate that patients were respiratory symptomatic and thus, presenting a higher probability of pulmonary TB.

The outcome of interest was time until death by any cause, with the exception of external causes (accidents, homicides and suicides). Deaths were identified through record linkage of cohort subjects to deaths during the follow-up period in the Mortality Information System of Pernambuco (SIM-PE), using the probabilistic linkage program RecLink III [20].

The main exposure of interest was empirical treatment for TB in the context of the study hypothesis as a protective factor for mortality in PLHIV. Treatment for pulmonary TB was initiated by the attending physician based on their clinical judgment. Antiretroviral therapy and TB treatment are distributed free of charge by the health services, and are restricted to cases notified to the System of Notification of Infectious Diseases (SINAN)/Pernambuco (PE).

Other explanatory variables were sex, age, socioeconomic status (individual monthly income, literacy and employment), and lifestyle (use of illicit drugs, alcohol and tobacco). Alcohol consumption, based on reported number of drinks per day, was classified according to the CDC [21], into abstainers, light drinkers and heavy drinkers (>two drinks/day for men and > one drink/day for women). Smoking was classified into: never smoker, former smoker (did not smoke at the time of the study nor during the preceding 6 months), and current smoker (smoked at the time of the study or quit less than six months before) [22].

Data was also collected on general health (Body Mass Index-BMI, anemia, loss of weight reported at baseline) and variables related with HIV and TB. HIV-related variables included CD4 T-cell count and opportunistic diseases during the previous three months. A combination of three different antiretroviral drugs was categorized as either using HAART or not, on entry to the cohort.

The CD4 T-cell count was measured during the follow-up period and was the only time-varying variable in the survival analysis, categorized as ≥ 200 cells/mm^3^ and < 200 cells/mm^3^. Variables related to TB included chest X-ray, history of previous TB treatment and bacteriology testing (whether negative or not performed).

Individuals were interviewed by a health professional using a tailored questionnaire, after signing the Statement of Informed Consent. Information regarding complaining of a cough, results of laboratory tests, and treatment for TB and HIV was abstracted from medical records using a specific form. Patients who reported a cough of any duration during the routine clinical visits were enrolled in this cohort.

### Statistical analysis

The follow-up period was considered from the time a cough was first registered until failure (death) or censoring: at the end of the study or on death from external causes (accidents, homicides or suicides). The Kaplan-Meier (KM) method was used to calculate the probability of survival for the whole study population and stratified for empirical treatment for TB (yes/no) and for CD4 cell count (≥ 200 cells/mm^3^ and < 200 cells/mm^3^. To identify factors associated with death we estimated the Hazard Ratio (HR) in bivariate and multivariate Cox regression analyses with 95% Confidence Intervals (CI) and p values to evaluate the statistical significance. We assessed the proportional-hazards assumption for each variable studied. Variables significantly associated with death in the bivariate analysis at a p value ≤ 0.20 were introduced into the multivariate model, according to their statistical significance and clinical and epidemiological importance. The final model retained variables associated with death at p value ≤ 0.05.

A second unplanned analysis was conducted to investigate an unexpected result: the higher mortality in those who received empirical treatment for TB. First, we investigated the characteristics of those who received empirical treatment (bivariate and multivariate logistic regression analyses) to verify whether these characteristics could explain the higher mortality. Secondly, we estimated the effect of the empirical treatment separately in those we considered less likely to have had TB and those more likely to have had TB, based on the presence of three indicators for the likelihood of TB: bacteriology testing, an abnormal chest X-ray and a history of previous TB treatment.

Our hypothesis was that empirical treatment for TB would only be associated with higher mortality rates in those unlikely to have had TB, proposing that treatment may have been introduced as a desperate measure given the severity of the HIV disease, (where mortality is very high). Data analysis was undertaken using STATA 11.2.

### Ethical approval

This study is part of the project “Clinical-epidemiological study of co-infection tuberculosis/HIV in Recife” which was approved by the Research Ethics Committee at the Universidade Federal de Pernambuco (CEP/CCS/UFPE 254/05).

## Results

**Figure 1:**
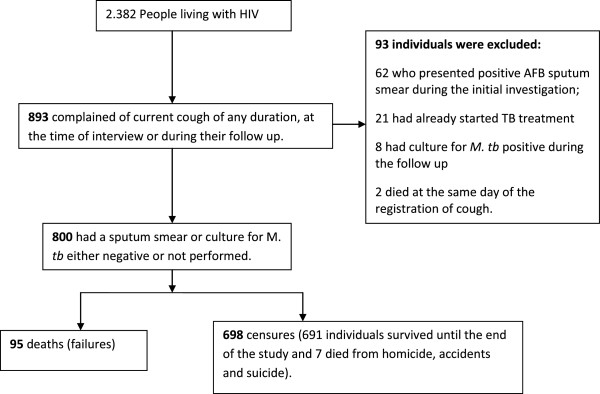
Flow chart of the study population.

From 2382 PLHIV attending the referral centers during the study period, 893 complained of a cough of any duration at any time during follow-up. Of these, 93 were excluded: 62 presented sputum smear positive, 21 had started TB treatment before registering a cough, eight presented a positive *M.tb* culture during the follow-up, and two died on the day a cough was recorded. The final analysis included 800 patients, with 698 censored, 691 surviving to the end of the study and 7 deaths from external causes and 95 deaths from clinical causes (failures) (Figure 1).

Table 1 shows the frequency distribution of demographic, socioeconomic and lifestyle factors. Most patients (59%) were male and 55% were aged under 40 years. Over 80% were literate and 47.5% had a monthly income ≥ 1 minimum wage (on average 170 dollars over the study period) and 25% were employed. Most were either current (40%) or former (23%) smokers. Past/current use of illicit drugs was reported by one in three patients.

**Table 1:** Frequency distribution and bivariate Cox regression analysis of the association between demographic, socioeconomic and lifestyle variables and death of PLHIV complaining of cough, Recife, 2010

	**N (%)**	**HR (CI 95%)**	***P*** **value**	**Proportionality test**
				***p value***
**Demographic variables**				
**Sex**				
Female	327 (40.9)	1.0		
Males	473 (59.1)	1.62 (1.0 – 2.5)	0.031	0.3761
**Age**				
**Age groups**				
< 40 years	437 (54.6)	1.0		
≥ 40 years	363 (45.4)	0.91 (0.6 – 1.4)	0.643	0.1676
**Socioeconomic variables**				
**Literate**				
Yes	678 (85.4)	1.0		
No	116 (14.6)	1.4 (0.8 – 2.3)	0.198	0.7981
**Individual monthly income (MW)**				
≥ 1	374 (47.5)	1.0		
< 1	413 (52.5)	1.1 (0.9 – 1.4)	0.272	0.2641
**Employment**				
Yes	191 (24.2)	1.0		
No	599 (75.8)	2.4 (1.3 – 4.6)	0.006	0.6009
**Lifestyle variables**				
**Alcohol consumption**				
Abstainer	497 (63.3)	1.0	0.003	
Light drinker	189 (24.1)	0.4 (0.2 - 0.7)		
Heavy drinker	99 (12.6)	0.6 (0.3 - 1.2)	0.193	0.3628
**Smoking status**				
Never smoker	296 (37.1)	1.0		
Former smoker	180 (22.6)	0.6 (0.3 - 1.2)	0.159	0.3839
Current smoker	322 (40.4)	1.4 (0.9 - 2.1)	0.170	
**Illicit drug use**				
Never	553 (69.5)	1.0		
Past or current	243 (30.5)	1.0 (0.6 - 1.5)	0.904	0.2202

Table 2 shows the distribution of general health variables, variables related to HIV and variables related to TB. At baseline, 62% had a BMI between 18.5 kg/m^2^ and 24.9 kg/m^2^ (eutrophic), 11% were underweight and 27% were overweight/obese; 45% had anemia and half reported opportunistic diseases in the three months before recruitment. During follow-up, almost all (99.4%) underwent at least one CD4 cell count measurement and 73.2% used HAART at entry. Previous TB treatment was reported by 22.7% and one hundred and seventy one (21%) individuals received empirical treatment for tuberculosis during follow-up. Bacteriology tests (AFB sputum smear or *M.tb* culture) were not performed in 61% (492) of the whole cohort, and this percentage dropped to 40.9% among the 171 patients who initiated presumptive TB treatment, during follow-up (data not shown). Half of the 492 people who did not undergo bacteriological testing was reported to have no sputum. Patients who did not undergo AFB sputum smear or *M.tb* culture testing during follow-up were similar to those who tested and were negative, in relation to sex, age, CD4 cell count and HAART at baseline (data not shown).

**Table 2:** Frequency distribution and bivariate Cox regression analysis of the association between general health, variables related to HIV and TB with death of PLHIV complaining of cough, Recife, 2010

	**N (%)**	**HR (CI 95%)**	***P*** **value**	**Proportionality test**
				***p value***
**General health variables**				
**BMI**				
18.5 to 24.9 kg/m^2^	483 (62.4)	1.0		
< 18.5 kg/m^2^	85 (11.0)	2.0 (1.1 – 3.4)	0.015	0.2341
≥ 25 kg/m^2^	206 (26.6)	0.6 (0.3 – 1.0)	0.056	
**Anemia**				
No	402 (54.6)	1.0		
Yes	334 (45.4)	5.6 (3.1 – 10.0)	0.000	0.9383
**Loss of weight reported at baseline**				
No	422 (56.1)	1.0		
Yes	330 (43.9)	2.2 (1.4 - 3.4)	0.000	0.3383
**Variables related to HIV**				
**HAART (baseline)**				
No	214 (26.8)	1.0		
Yes	585 (73.2)	1.3 (0.8 - 2.1)	0.299	0.0055
**CD4 cell count (time varying)**				
≥ 200	-	1.0		
< 200	-	9.1 (5.9 – 14.0)	0.000	0.5200
**Opportunistic disease reported at baseline**				
No	405 (50.6)	1.0		
Yes	395 (49.4)	2.0 (1.3 – 3.0)	0.001	0.1302
**Variables related to tuberculosis**				
**AFB sputum smear or *M. Tb *** **culture during follow-up**				
Not performed	492 (61.5)	1.0		
Negative	308 (38.5)	1.7 (1.1 – 2.5)	0.010	0.9815
**Chest X-ray**				
Normal	364 (45.5)	1.0		
Abnormal	154 (19.3)	4.0 (2.4 - 6.6)	0.000	
Not performed	282 (35.3)	1.7 (1.0 - 2.9)	0.045	0.0301
**Empirical TB treatment during follow-up**				
No	629 (78.6)	1.0		
Yes	171 (21.4)	7.3 (4.8 – 11.0)	0.000	0.8524
**History of previous TB treatment**				
No	618 (77.3)	1.0		
Yes	182 (22.7)	1.9 (1.2 - 2.9)	0.003	0.6722

**Figure 2:**
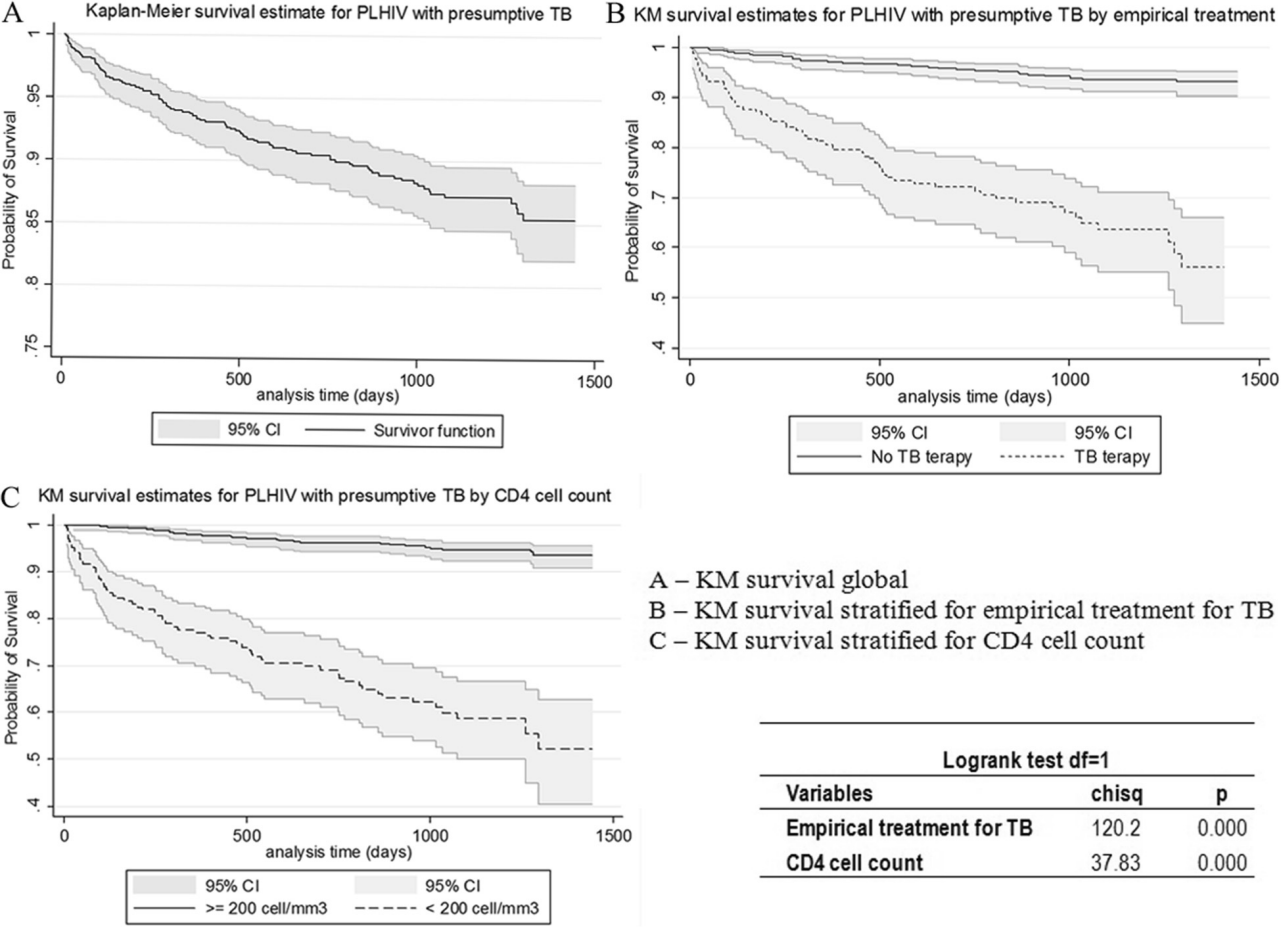
**Kaplan Meier estimate for survival probability of PLHIV with Presumptive TB. A)** KM estimate for survival probability of the whole study population with 95% CI and logrank test. **B)** KM estimate for survival probability stratified by empirical treatment for tuberculosis with 95% CI and logrank test. **C)** KM estimate for survival probability stratified by CD4 cell count with 95% CI and logrank test.

The mortality rate was 4.6 per 100 py. Figure 2 shows the Kaplan-Meier 85.4% probability of survival by the end of the follow-up period (3.9 years), the KM for empirical TB treatment (Yes= 56.2% and No= 93.1%) and the KM for CD4 < 200 cells/mm^3^ (52.2%) and CD4 ≥ 200 cells/mm^3^ (93.6%).

The median time of follow-up was 2.8 years. The mortality rate among those with no bacteriology testing was 3.4 per 100 py and among those who presented negative bacteriology tests 6.4 per 100 py. Mortality was higher in those who underwent empirical treatment (16.1 per 100 py), than for those who did not (2.1 per 100 py). The average time from registering a cough until TB treatment was almost six months, and was greater for those who died (183 days) than for those who did not (153 days). However, this was not statistically significant (p = 0.499). We verified that those who did not report a history of previous TB treatment and those who had normal chest X-rays presented a greater average time from the registration of a cough until TB treatment compared with those who reported a history of previous TB treatment and those with abnormal chest X-rays, respectively, and the differences were statistically significant. There was no difference in the average time from registering a cough until TB treatment between patients who did not undergo AFB sputum smear or *M.tb* culture testing during follow-up and those who tested negative.

Tables 1 and 2 demonstrate the crude association between death and the demographic, socioeconomic and lifestyle variables; variables related to general health, as well as those related to HIV and TB, which were statistically significant at a p value ≤ 0.20.

Table 3 illustrates the final adjusted model. Risk of death was higher in those with anemia (HR = 2.8), during periods of low CD4 count (HR = 5.1), for those with an abnormal chest X-ray (HR = 1.9) and those receiving empirical treatment for TB (HR = 3.7). Light drinkers presented a lower risk of death compared to abstainers. The proportional-hazards assumption in the final model was respected (phtest = 0.7073).

**Table 3:** Multivariate Cox regression analysis of the association between the variables studied and death of PLHIV complaining of cough, Recife, 2010

	**N (%)**	**HR (CI 95%)**	***P*** **value**
**Alcohol consumption**			
Abstainer	497 (63.3)	1.0	
Light drinker	189 (24.1)	0.4 (0.2 - 0.9)	0.049
Heavy drinker	99 (12.6)	1.0 (0.5 - 1.9)	0.907
**Anemia**			
No	402 (54.6)	1.0	
Yes	334 (45.4)	2.8 (1.5 - 5.3)	0.001
**CD4 cell count (time varying)**			
≥ 200	---	1.0	
< 200		5.1 (3.1 – 8.6)	0.000
**Chest X-ray**			
Normal	364 (45.5)	1.0	
Abnormal	154 (19.3)	1.9 (1.1 – 3.3)	0.001
Not performed	282 (35.3)	1.5 (0.8 – 2.7)	0.146
**Empirical TB treatment during follow-up**			
No	629 (78.6)	1.0	
Yes	171 (21.4)	3.7 (2.2 – 6.3)	0.000

Phtest (p = 0.7073).

Table 4 compares those who did and did not receive empirical treatment for TB. Treated subjects were more likely to have characteristics suggestive of severe HIV disease (low CD4, opportunistic infections) and characteristics suggestive of tuberculosis (abnormal chest X-ray, history of TB treatment, bacteriology testing) and characteristics suggestive of both TB and severe HIV: reported weight-loss and anemia.

**Table 4:** Multivariate Logistic regression analysis of the association between the variables studied and empirical TB treatment for PLHIV complaining of cough, Recife, 2010

	**Odds ratio (IC 95%)**	***p***
**CD4 cell count (time varying)**		
≥ 200 cel/mm^3^	1.0	
**<** 200 cel/mm^3^	3.0 (1.8 – 4.8)	0.000
**Anemia**		
No	1.0	
Yes	3.4 (2.1 – 5.5)	0.000
**Loss of weight reported at baseline**		
No	1.0	
Yes	1.9 (1.2 – 3.0)	0.008
**Opportunistic disease reported at baseline**		
No	1.0	
Yes	2.0 (1.2 – 3.4)	0.005
**History of previous TB treatment**		
No	1.0	
Yes	2.3 (1.4 – 3.8)	0.002
**Chest X-ray**		
Normal	1.0	
Abnormal	2.0 (1.1 – 3.5)	0.016
No performed	0.72 (0.4 – 1.3)	0.253
**Sputum smear ABF or *M. tuberculosis *** **culture during follow-up**		
No	1.0	
Negative	2.4 (1.5 - 3.8)	0.000

It is not demonstrated in the table that 74% of patients with the three characteristics suggestive of TB received empirical treatment, and in this group mortality was not significantly higher in those treated (HR = 1.5; p = 0.590). Less than 10% of those with none of the 3 characteristics suggestive of TB received empirical treatment, which in this group was significantly associated with a marked increase in mortality (HR = 14.3; p <0.001).

## Discussion

The mortality rate encountered in our study, 4.6 per 100 person-years, was lower than in similar populations in Zimbabwe (17.9/100py) [17] and South Africa (28.9 per 100 py) [23]. This could be due to a more frequent use of HAART (73.2% in our study compared to 21.3% in SA). HAART reduces case fatality in smear-negative pulmonary TB [15].

Those who underwent bacteriology testing presented a higher mortality rate (1.7 higher) when compared with those who did not, although this was not significant in the multivariate model. The request for bacteriology tests may reflect the strength of the attending physician’s suspicion of tuberculosis, and indicate a higher likelihood of tuberculosis.

The highest increase in mortality occurred when patients presented a low CD4 cell count (< 200). This is consistent with the literature: advanced immunosuppression was the main risk factor for death in PLHIV with negative bacteriology tests and presumptive TB [17].

Chest X-ray investigation is recommended soon after a cough to shorten delays in diagnosis and treatment of smear negative pulmonary TB in PLHIV [1,24]. In our study, 64.8% of the patients performed a chest X-ray, of which one in three was abnormal. Abnormalities were associated with empirical treatment for TB and were a predictor of death, again in our view, indicating a higher likelihood of tuberculosis.

We studied smoking, alcohol consumption, and illicit drug use because these factors have been associated with either TB/HIV disease progression or death in PLHIV [25-28]. All three were very frequent in our study population but none were associated with death in the multivariate model. Abstemious subjects presented higher mortality, which in our view, is possibly a consequence of ill health.

Contrary to our expectations, mortality was higher in those who received empirical treatment for TB. If all other factors were the same, we would expect that in patients with cough (used in the study to indicate that they are respiratory symptomatic and thus, with a higher probability of pulmonary TB), empirical TB treatment would have improved prognosis [3,15-17], particularly in this population with a high HAART uptake since the start of follow-up [3,6].

To explore this further, we investigated characteristics of those receiving empirical treatment: they had both more characteristics suggesting TB and severe HIV disease. Mortality was not significantly higher in those treated with three characteristics suggestive of TB (abnormal chest X-ray, history of previous TB treatment, AFB sputum smear or *M.tb* culture testing).

In those without the three characteristics strongly suggestive of TB, mortality was 14 times higher in those receiving empirical treatment. This is consistent with the attending physician being more likely to introduce tuberculosis treatment as a final resource in patients more likely to die. This could either be because there was another cause for the cough or because of severe HIV disease, or both. Empirical treatment was more frequent with a lower CD4 count, anemia, weight loss and those who reported past opportunistic disease. A greater percentage (44.6%) of patients with a CD4 < 200 was treated empirically for TB compared with only 15.5% of patients with a CD4 cell count ≥ 200 (data not shown). Although we performed a control for other predictors of mortality, such as anemia, weight loss, and a low CD4 count, the occurrence of residual confounding is very likely as the measurements were not conducted very frequently: for example, on average, a CD4 count was measured every 6 months. In a similar cohort it would be important to ascertain that other potential diagnoses that may mimic TB had not been overlooked [3]. Immune reconstitution syndrome could have caused death due to unmasked TB after the initiation of HAART. However, the impact of this on the mortality rate in this cohort was probably diminished by the small number of patients who had used HAART for less than one year.

Empirical treatment for TB may have been introduced too late to prevent death, especially among those patients who presented normal chest X-rays and did not report a history of previous TB treatment. These findings reinforce the need to speed up TB diagnosis and treatment among those with presumptive TB and AFB sputum smear negative [19].

At the time of data collection there was no internationally accepted symptoms score system based on evidence for diagnosing negative sputum pulmonary TB in PLHIV [29], and treatment, based on clinical judgment, was introduced on average, 6 months after a cough was registered. To identify an optimum standardized rule for TB screening among PLHIV, a meta-analysis of observational studies found that the presence of at least one of the symptoms: cough of any duration, fever, night sweats and weight loss, had a sensitivity of 78.9% to identify TB in PLHIV. Abnormal chest X-ray findings increased sensitivity by 11% [29]. This evidence underlies the WHO recommendation to introduce screening for TB in the routine care of PLHIV [19].

One limitation of our study concerns that of observational studies to evaluate the effectiveness of empirical treatment for TB, which was initiated by physicians during routine care within the health services. Besides this, the empirical treatment for TB did not follow a well-defined protocol for screening TB, which would have ensured the early introduction of TB treatment, and also avoid the misdiagnosis of other opportunistic diseases that could present with cough. Furthermore, data on opportunistic diseases were collected only at baseline and not during the follow-up period.

Observation studies on empirical TB treatment should be used to evaluate the processes involved, e.g., prescribing practices, completeness of treatment, but not to evaluate the impact of empirical treatment for TB, since there is very strong residual confounding and the impact cannot be evaluated accurately. Even so, we believe that it is very important to reveal our results because, probably, this is a very common situation in many settings, especially in the developing world where the presumptive diagnosis of TB in PLHIV occurs extensively.

## Conclusions

In this routine setting, the large majority of PLHIV with strong indications of presenting with tuberculosis were receiving empirical treatment, although late. Their mortality was still a little higher than those with strong indications of TB who did not receive treatment, but this could result from the stronger likelihood for them to present with tuberculosis. Mortality was not significantly higher in those patients empirically treated for TB, who presented three characteristics suggestive of the disease (abnormal chest X-ray, history of TB treatment, AFB sputum smear or *M.tb* culture testing). Those with strong indications of advanced HIV diseases were more likely to receive empirical TB treatment (even when presenting fewer indications of tuberculosis); those receiving treatment in this group had a marked increase in mortality, which most likely resulted from their advanced HIV disease, and possibly from other causes for the cough. Although we performed a control for the CD4 cell count in the analysis, we believe that severe HIV disease was a very strong confounder of the association between empirical TB treatment and mortality.

## Competing interests

The authors declare that they have no competing interests.

## Authors’ contributions

MFPMA conceived and designed the study. MFPMA, IC, JDLB, MM, RAAX, HLR and DBMF performed the study. IC and JDLB prepared the database. MFPMA, RAAX, JDLB and LCR analyzed the data. MFPMA, RAAX, HLR and DBMF contributed reagents/materials/analysis tools. MFPMA, RAAX and LCR wrote the paper. LCR discussed the study design and participated in the analysis. All authors read and approved the final manuscript.

## Pre-publication history

The pre-publication history for this paper can be accessed here:

http://www.biomedcentral.com/1471-2458/14/289/prepub

## References

[B1] World Health Organization, UNAIDS, UNICEF Global HIV/AIDS Response: Epidemic Update and Health Sector Progress Towards Universal Access: Progress Report 2011 2011 Geneva: Switzerland: WHO Press

[B2] Braitstein P Brinkhof MW Dabis F Schechter M Boulle A Miotti P Wood R Laurent C Sprinz E Seyler C Bangsberg DR Balestre E Sterne JA May M Egger M Mortality of HIV-1-infected patients in the first year of antiretroviral therapy: comparison between low-income and high-income countries Lancet 2006 367 817 824 16530575 10.1016/S0140-6736(06)68337-2

[B3] Lawn SD Ayles H Egwaga S Williams B Mukadi YD Santos Filho ED Godfrey-Faussett P Granich RM Harries AD Potential utility of empirical tuberculosis treatment for HIV-infected patients with advanced immunodeficiency in high TB-HIV burden settings Int J Tuberc Lung Dis 2011 15 287 295 21333094

[B4] Bradshaw D Laubscher R Dorrington R Bourne DE Timaeus IM Unabated rise in number of adult deaths in South Africa S Afr Med J 2004 94 278 279 15150940

[B5] Lawn SD Harries AD Anglaret X Myer L Wood R Early mortality among adults accessing antiretroviral treatment programmes in sub-Saharan Africa AIDS 2008 22 1897 1908 10.1097/QAD.0b013e32830007cd 18784453 PMC3816249

[B6] World Health Organization Treatment of Tuberculosis: Guidelines for National Programmes 2010 Geneva: Switzerland: WHO

[B7] Grinsztejn B Veloso VG Friedman RK Moreira RI Luz PM Campos DP Pilotto JH Cardoso SW Keruly JC Moore RD Early mortality and cause of deaths in patients using HAART in Brazil and the United States AIDS 2009 23 2107 2114 10.1097/QAD.0b013e32832ec494 19770698 PMC3790467

[B8] Corbett EL Marston B Churchyard GJ De Cock KM Tuberculosis in sub-Saharan Africa: opportunities, challenges, and change in the era of antiretroviral treatment Lancet 2006 367 926 937 10.1016/S0140-6736(06)68383-9 16546541

[B9] Lopez-Gatell H Cole SR Margolick JB Witt MD Martinson J Phair JP Jacobson LP Effect of tuberculosis on the survival of HIV-infected men in a country with low tuberculosis incidence AIDS 2008 22 1869 1873 10.1097/QAD.0b013e32830e010c 18753866 PMC3079345

[B10] Manosuthi W Chottanapand S Thongyen S Chaovavanich A Sungkanuparph S Survival rate and risk factors of mortality among HIV/tuberculosis-coinfected patients with and without antiretroviral therapy J Acquir Immune Defic Syndr 2006 43 42 46 10.1097/01.qai.0000230521.86964.86 16885778

[B11] Sanguanwongse N Cain KP Suriya P Nateniyom S Yamada N Wattanaamornkiat W Sumnapan S Sattayawuthipong W Kaewsa-ard S Ingkaseth S Varma JK Antiretroviral therapy for HIV-infected tuberculosis patients saves lives but needs to be used more frequently in Thailand J Acquir Immune Defic Syndr 2008 48 181 189 10.1097/QAI.0b013e318177594e 18520676

[B12] Velasco M Castilla V Sanz J Gaspar G Condes E Barros C Cervero M Torres R Guijarro C Effect of simultaneous use of highly active antiretroviral therapy on survival of HIV patients with tuberculosis J Acquir Immune Defic Syndr 2009 50 148 152 10.1097/QAI.0b013e31819367e7 19131895

[B13] Abdool Karim SS Naidoo K Grobler A Padayatchi N Baxter C Gray A Gengiah T Nair G Bamber S Singh A Khan M Pienaar J El-Sadr W Friedland G Abdool Karim Q Timing of initiation of antiretroviral drugs during tuberculosis therapy N Engl J Med 2010 362 697 706 10.1056/NEJMoa0905848 20181971 PMC3076221

[B14] Hanifa Y Fielding KL Charalambous S Variava E Luke B Churchyard GJ Grant AD Tuberculosis among adults starting antiretroviral therapy in South Africa: the need for routine case finding Int J Tuberc Lung Dis 2012 16 1252 1259 10.5588/ijtld.11.0733 22794030

[B15] Getahun H Harrington M O’Brien R Nunn P Diagnosis of smear-negative pulmonary tuberculosis in people with HIV infection or AIDS in resource-constrained settings: informing urgent policy changes Lancet 2007 369 2042 2049 10.1016/S0140-6736(07)60284-0 17574096

[B16] Harries AD Paying attention to tuberculosis suspects whose sputum smears are negative Int J Tuberc Lung Dis 2011 15 427 428 10.5588/ijtld.11.0051 21396196

[B17] Macpherson P Dimairo M Bandason T Zezai A Munyati SS Butterworth AE Mungofa S Rusakaniko S Fielding K Mason PR Corbett EL Risk factors for mortality in smear-negative tuberculosis suspects: a cohort study in Harare, Zimbabwe Int J Tuberc Lung Dis 2011 15 1390 1396 10.5588/ijtld.11.0056 22283900 PMC3272461

[B18] Ministry of Health, Secretaria de Vigilância em Saúde Manual e recomendações para o controle da tuberculose no Brasil 2011 Brazil: Ministério da Saúde 284 [in Portuguese]

[B19] World Health Organization Guidelines for Intensified Tuberculosis Case-Finding and Isoniazid Preventive Therapy for People Living with HIV in Resource-Constrained Settings 2011 Geneva: Switzerland: WHO

[B20] Camargo KR Jr Coeli CM [Reclink: an application for database linkage implementing the probabilistic record linkage method] Cad Saude Publica 2000 16 439 447 10.1590/S0102-311X2000000200014 10883042

[B21] CDC Fact Sheets in Alcohol and Public Health 2010 Atlanta, GA: Centers for Disease Control and Prevention

[B22] Ariyothai N Podhipak A Akarasewi P Tornee S Smithtikarn S Thongprathum P Cigarette smoking and its relation to pulmonary tuberculosis in adults Southeast Asian J Trop Med Public Health 2004 35 219 227 15272772

[B23] Benova L Fielding K Greig J Nyang’wa BT Casas EC da Fonseca MS du Cros P Association of BMI category change with TB treatment mortality in HIV-positive smear-negative and extrapulmonary TB patients in Myanmar and Zimbabwe PloS one 2012 7 4 e35948 10.1371/journal.pone.0035948 22545150 PMC3335812

[B24] World Health Organization Improving the diagnosis and treatment of smear-negative pulmonary and extra-pulmonary tuberculosis among adults and adolescents. Recommendations for HIV-prevalent and resource-constrained settings WHO/HTM/HIV 2007 Geneva, Switzerland: WHO

[B25] Raviglione M Marais B Floyd K Lonnroth K Getahun H Migliori GB Harries AD Nunn P Lienhardt C Graham S Chakaya J Weyer K Cole S Kaufmann SH Zumla A Scaling up interventions to achieve global tuberculosis control: progress and new developments Lancet 2012 379 1902 1913 10.1016/S0140-6736(12)60727-2 22608339

[B26] Slama K Chiang CY Enarson DA Hassmiller K Fanning A Gupta P Ray C Tobacco and tuberculosis: a qualitative systematic review and meta-analysis Int J Tuberc Lung Dis 2007 11 1049 1061 17945060

[B27] Neuman MG Schneider M Nanau RM Parry C Alcohol consumption, progression of disease and other comorbidities, and responses to antiretroviral medication in people living with HIV AIDS Res Treat 2012 2012 751827 22496971 10.1155/2012/751827 PMC3310201

[B28] Duailibi LB Ribeiro M Laranjeira R Profile of cocaine and crack users in Brazil Cad Saude Publica 2008 24 Suppl 4 s545 s557 18797730 10.1590/s0102-311x2008001600007

[B29] Getahun H Kittikraisak W Heilig CM Corbett EL Ayles H Cain KP Grant AD Churchyard GJ Kimerling M Shah S Lawn SD Wood R Maartens G Granich R Date AA Varma JK Development of a standardized screening rule for tuberculosis in people living with HIV in resource-constrained settings: individual participant data meta-analysis of observational studies PLoS Med 2011 8 e1000391 10.1371/journal.pmed.1000391 21267059 PMC3022524

